# Pulmonary sinus cusp–inclusive mapping and ablation for paediatric right ventricular outflow tract ventricular arrhythmias

**DOI:** 10.1093/europace/euag107

**Published:** 2026-05-07

**Authors:** Yi-Xiang Lin, Zhi-Yu Feng, Lu Zhao, Xue-Cun Liang, Qiu-Ping Lin, Qu-Ming Zhao

**Affiliations:** Paediatric Heart Center, National Children’s Medical Center, Children’s Hospital of Fudan University, 399 Wanyuan Road, Shanghai, China; Paediatric Heart Center, National Children’s Medical Center, Children’s Hospital of Fudan University, 399 Wanyuan Road, Shanghai, China; Paediatric Heart Center, National Children’s Medical Center, Children’s Hospital of Fudan University, 399 Wanyuan Road, Shanghai, China; Paediatric Heart Center, National Children’s Medical Center, Children’s Hospital of Fudan University, 399 Wanyuan Road, Shanghai, China; Paediatric Heart Center, National Children’s Medical Center, Children’s Hospital of Fudan University, 399 Wanyuan Road, Shanghai, China; Paediatric Heart Center, National Children’s Medical Center, Children’s Hospital of Fudan University, 399 Wanyuan Road, Shanghai, China

**Keywords:** Right ventricular outflow tract, Ventricular arrhythmias, Radiofrequency ablation, Pulmonary sinus cusps, Children

## Introduction

Catheter ablation for idiopathic right ventricular outflow tract (RVOT) ventricular arrhythmias (VAs) is usually effective in children, but procedures can become difficult when ectopy is suppressed under general anaesthesia and catheter contact is unstable along the high RVOT free wall.^1,2^ Adult studies support the pulmonary sinus cusps (PSCs) as actionable targets for RVOT-type VAs, but paediatric data remain limited.^3–5^ We prospectively evaluated a PSC-inclusive workflow in children, emphasizing low-ectopy procedures, PSC-subvalvular mapping relationships, and intracuspal target distribution.

## Methods

**Figure 1:**
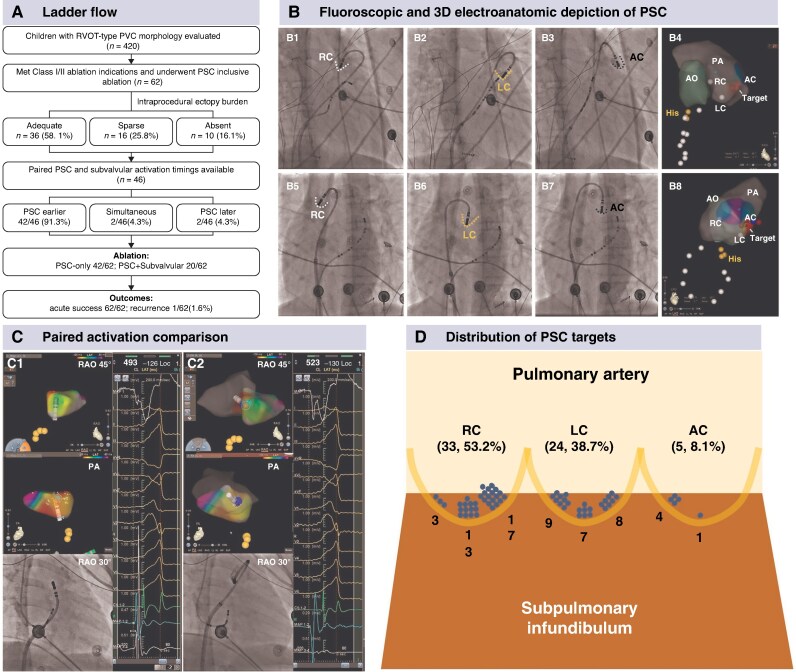
(*A*) Ladder flow of the study cohort. (*B*) Fluoroscopic and three-dimensional electroanatomic depiction of PSCs. B1–B3 (RAO 30°) and B5–B7 (LAO 45°) show hand-injected contrast outlining the right, left, and anterior cusps, indicated by dashed lines. B4 and B8 show the corresponding CARTO™ reconstruction. AC, anterior cusp; AO, aorta; His, His-bundle region; LAO, left anterior oblique; LC, left cusp; PA, pulmonary artery; PSC, pulmonary sinus cusp; RC, right cusp; RAO, right anterior oblique. (*C*) Representative paired activation mapping comparing subvalvular vs. supravalvular earliest activation. C1 Subvalvular mapping identified the earliest site in the posterior septal right ventricular outflow tract (tag; LAT −126 ms). C2 PSC mapping identified an earlier supravalvular earliest site within the right cusp (tag; LAT −130 ms). Although the two sites were in close proximity, the supravalvular site showed sharper, higher-quality unipolar and bipolar electrograms, supporting PSC precedence. LAT, local activation time. (*D*) Schematic illustration of the distribution of successful PSC ablation sites. Dots represent effective targets, and numbers denote counts within each segment.

This was a prospective single-centre cohort study at Children’s Hospital of Fudan University (July 2023–December 2025) (*Figure 1A*). Inclusion required an RVOT-type 12-lead ECG pattern and Class I or II paediatric ablation indications (symptoms, VA-related ventricular dilation or dysfunction, or exercise-related increase in VA burden or complexity).^6^ Cases were excluded if intraprocedural mapping localized the arrhythmia to low RVOT, left ventricular outflow tract, intramural, or epicardial sites.

Procedures were performed under general anaesthesia using CARTO 3 and an irrigated B-curve, non–contact-force ThermoCool™ catheter. Supravalvular positioning was confirmed by hand injection of contrast through the ablation catheter irrigation lumen (*Figure 1B*) and transthoracic echocardiography. Intraprocedural VA burden was categorized as adequate [at least one premature ventricular contraction（PVC)/min or inducible/sustained VA], sparse (less than one PVC/min), or absent.^7^ When ectopy was limited, pace mapping was performed using a predefined protocol (starting at 2 mA/2 ms and escalating if needed).^8^

After subvalvular mapping defined a candidate high-RVOT sector, systematic PSC interrogation was performed in the corresponding cusp region, and PSC lesions were delivered first; adjunct subvalvular lesions were added when PSC precedence was indeterminate or when subvalvular signals were equivalent or earlier. Acute success required elimination of the clinical VA and non-inducibility after a 30-min waiting period; in sparse/absent ectopy cases, loss of local capture at the final PSC target despite high-output pacing (20 mA/2 ms) was prespecified as an adjunctive endpoint. For left-cusp RF delivery, safety assessment relied on continuous ECG monitoring for ischaemic ST-T changes and conservative energy delivery (25 W for 60 s in the left cusp; 30 W for 60–90 s elsewhere). Follow-up included ECG, 24-h Holter, and echocardiography at 1 day; 1, 3, 6, and 12 months; and annually thereafter. Statistical analyses were performed using SPSS 26.0.

## Results

During the study period, 420 children with RVOT-type PVC morphology were evaluated, and 62 underwent ablation. The cohort had a mean age of 9.9 ± 3.0 years, and 35/62 (56.5%) were male. Mean baseline VA burden was 23.0 ± 8.4%.

Intraprocedural ectopy was adequate in 36/62 (58.1%), sparse in 16/62 (25.8%), and absent in 10/62 (16.1%). Activation mapping was feasible in the PSCs in 48/62 (77.4%) and in the subvalvular RVOT in 52/62 (83.9%). Among analysable cases, earliest activation preceded QRS by 38.6 ± 8.8 ms in the PSCs and 30.8 ± 7.8 ms at subvalvular RVOT sites. Paired PSC and subvalvular activation timings were available in 46 patients: PSC activation was earlier in 42/46 (91.3%), simultaneous in 2/46 (4.3%), and later in 2/46 (4.3%) (*Figure 1A, C*).

All patients underwent PSC interrogation and PSC-first ablation. After PSC-first ablation, no further clinical VA was observed or inducible. Adjunct subvalvular lesions were applied in 20/62 (32.3%) for consolidation when PSC precedence could not be established (e.g. sparse/absent ectopy) or when an equivalent/better subvalvular target was recorded. At the final PSC target, local capture was present before ablation and absent after ablation in all cases. Effective PSC targets clustered mainly near cusp borders/commissural regions rather than the central cusp floor (*Figure 1D*).

Mean procedure duration was 50 ± 15 min, fluoroscopy time 47.8 ± 8.7 s, dose–area product 1.6 ± 0.8 Gy·cm^2^, and total RF time 205 ± 32 s. Acute success was achieved in all 62 procedures. During follow-up (median 16 months), recurrence occurred in 1/62 (1.6%), within 2 months after ablation. No vascular complications, pericardial effusion, intraprocedural ST-T changes suggestive of acute coronary ischaemia, or post-procedural clinical findings of coronary injury were observed. No patient had more than mild pulmonary regurgitation before ablation; two developed new mild pulmonary regurgitation on predischarge echocardiography, without progression during follow-up.

## Discussion

This study adds prospective paediatric data on a PSC-inclusive workflow, highlighting its utility in low-ectopy procedures, its relationship to adjacent subvalvular mapping, and the distribution of successful PSC targets. Systematic PSC interrogation provided a reproducible supravalvular option where subvalvular catheter stability can be limited and remained applicable in a substantial proportion of cases with sparse or absent intraprocedural ectopy.

Several mechanisms may explain the efficacy of PSC ablation. Earlier supravalvular activation supports involvement of myocardial extensions above the pulmonary valve, but should not be interpreted as proof that the true site of origin lies within the PSCs. Rather, the PSCs and adjacent high subvalvular RVOT likely form a functional continuum in which the earliest actionable site may sometimes be more accessible from above the valve. In that setting, the cusp base may provide a more stable vantage point for pacing and lesion delivery when subvalvular contact is limited.^9^ Supravalvular and subvalvular pace maps may also appear similar because both can reflect the exit rather than the true source,^10^ helping explain why adjunct subvalvular lesions remained necessary in some cases.

Clinically, PSC interrogation may be particularly useful when catheter stability along the RVOT free wall is limited, when ectopy is sparse or absent under general anaesthesia, or when subvalvular pace maps are discordant or borderline. In low-ectopy procedures, standard provocation remains essential, and loss of local capture should be interpreted only as a prespecified adjunctive endpoint.

This study has limitations. It is a single-centre experience from a high-volume referral programme with dedicated PSC expertise, and the sample size is modest. We did not perform a formal ECG comparison between PSC and subvalvular VA patterns, because the study was not designed to define the true site of origin. Intracardiac echocardiography and routine coronary angiography were not performed.

In conclusion, a PSC-inclusive mapping and ablation workflow was feasible and safe, with high acute success and low recurrence. Pulmonary sinus cusp interrogation appears particularly useful when ectopy is limited under general anaesthesia or when subvalvular catheter stability is suboptimal.

## Data Availability

The data underlying the findings of this article are available upon reasonable request to the corresponding author.
